# Kawasaki Disease: An Unusual Case of a Retrospective Diagnosis in a 39-Year-Old Gentleman With Coronary Ectasias

**DOI:** 10.7759/cureus.11616

**Published:** 2020-11-22

**Authors:** Abbas Ali Khan, Faryal M Cheema, Saad Ahmad, Tahir Nazir, Irfan M Ahmed

**Affiliations:** 1 Cardiology, Royal Preston Hospital, Preston, GBR; 2 Cardiology, Royal Lancaster Infirmary, Lancaster, GBR; 3 Internal Medicine, Royal Preston Hospital, Preston, GBR

**Keywords:** kawasaki, coronary, aneurysm, ectasia

## Abstract

Kawasaki disease (KD) was first reported by Dr. Tomisaku Kawasaki in 1967 and is classified as an autoimmune vasculitis of small- and medium-sized arteries. It is usually a self-limited condition occurring in childhood, but it can have complications such as coronary artery aneurysms, myocardial ischemia, and arrhythmias with significant morbidity and mortality presenting later in life. We report a case of a KD presenting in adulthood with late cardiovascular sequelae managed with coronary artery bypass graft.

## Introduction

Kawasaki disease (KD) is a vasculitis, of uncertain etiology involving small- and medium-sized arteries including the coronary arteries [1]. Typically, it is a self-limiting condition that affects children less than five years of age, but there are long-term sequelae of the disease that can cause significant morbidity in adulthood. It is a rare but important differential in young patients presenting with chest pain, as illustrated in our case.

## Case presentation

**Figure 1:**
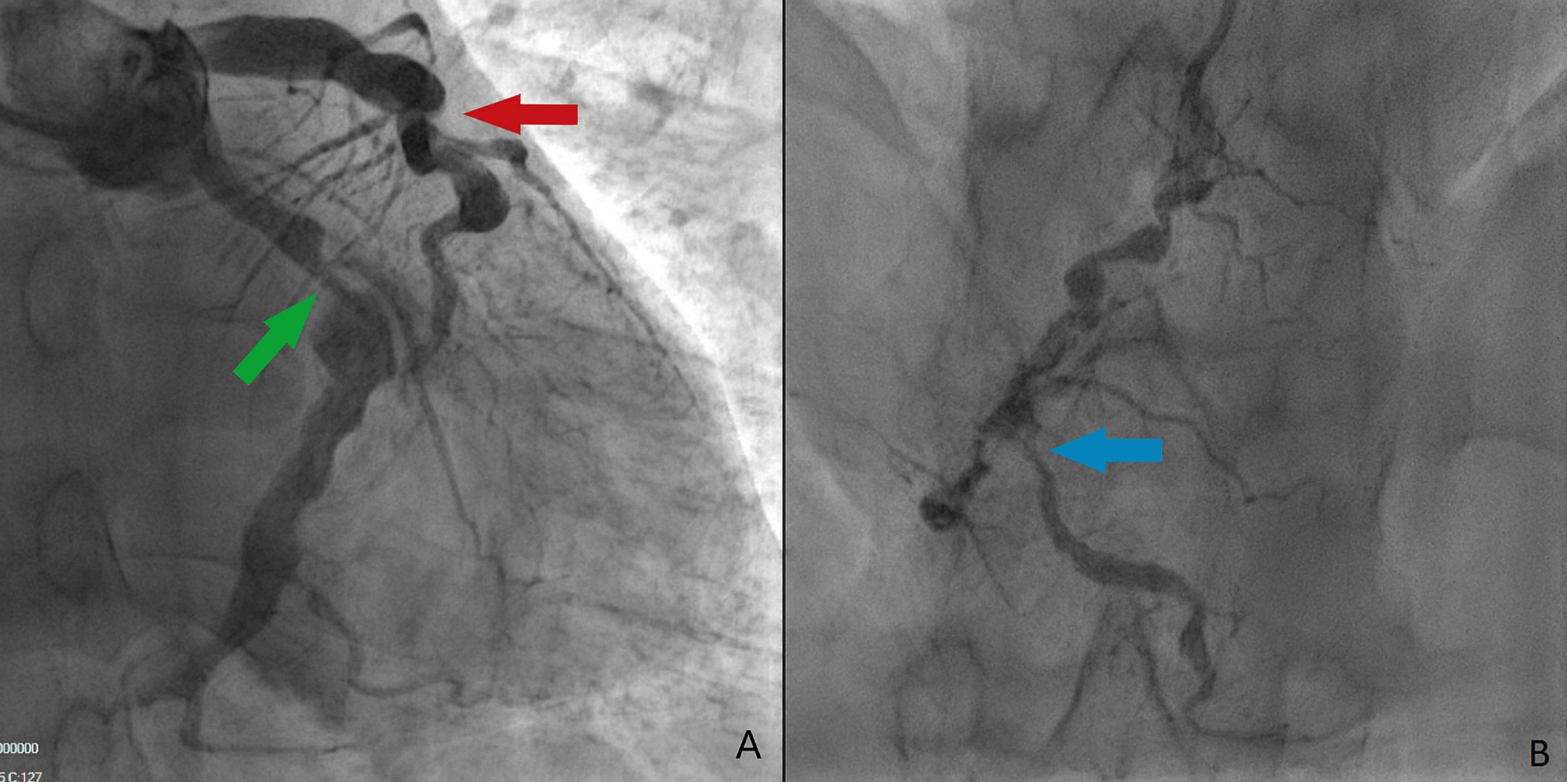
Initial coronary angiogram. A: Left-sided arteries; green arrow indicating 70% stenosis in LCX, red arrow indicating highly ectatic LAD B: Ectatic RCA with mid-portion stenosis (blue arrow) LCX, left circumflex; LAD, left anterior descending artery; RCA, right coronary artery

A 39-year-old South Asian male presented to the Royal Preston Hospital, Preston, Lancashire with burning central chest pain in 2017. There was no associated radiation, palpitations, shortness of breath, or any signs of hemodynamic instability. He was a smoker and had a history of familial hypercholesterolemia. Of note, this patient had a history of pyrexia of unknown origin in his infancy. His family history included Kawasaki disease, with his niece having been diagnosed with the disease. Preliminary investigations in the ED showed evidence of non-ST elevation myocardial infarction (NSTEMI) and he was transferred to the local percutaneous coronary intervention (PCI) centre for further investigation. His coronary angiogram in 2017 (Figure 1) revealed unusual coronary anatomy with significant coronary ectasia. There was 70% stenosis of the left circumflex (LCX) with a dilated and aneurysmal, thought to be the culprit lesion and a mid-right coronary artery lesion. There were multiple aneurysms and ectatic segments throughout the coronary vasculature.

A diagnosis of KD was made based on these findings. A dobutamine stress echo was performed which did not show any evidence of inducible ischemia (Video 1). He was treated conservatively but this proved to be troublesome as his compliance with medications was poor.

**Video 1:** Dobutamine stress echo; apical four chamber view showing no evidence of inducible ischemia.

**Figure 2:**
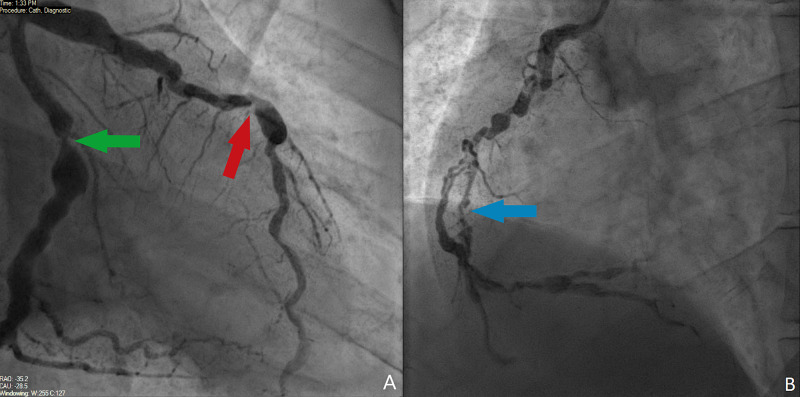
Repeat coronary angiography. A: Left-sided coronary arteries; LCX artery with 95% stenosis (green arrow) in the mid-portion with diffuse ectasia throughout and LAD with 75% stenosis in the mid vessel (red arrow) B: Right-sided coronary artery with 90% stenosis (blue arrow) and diffuse ectasia LCX, left circumflex; LAD, left anterior descending artery

He presented to the ED again with a prolonged episode of central chest pain. On examination, he had normal heart sounds, with equal pulses bilaterally, clear chest, and no signs of heart failure. It was noticed that he had bilateral xanthelasma and tendon xanthomas. His electrocardiogram showed no ischemic changes, however, troponins were elevated with serial samples measuring at 33, 26, and 18 ng/L. He was treated for NSTEMI again in 2019. Repeat angiogram in 2019 (Figure 2) revealed the left anterior descending artery (LAD) had diffuse ectatic disease with 75% stenosis in the mid-portion measuring 13 mm, the LCX had diffuse ectatic disease with 95% stenosis in the mid-portion and ectatic disease in the right coronary artery (RCA) with 90% stenosis in the mid-portion measuring 18 mm. The left main steam was unobstructed.

A CT-Thorax was also done which showed normal carotid arteries and aorta. A dobutamine stress echocardiogram showed viable myocardium. Given the findings of triple vessel disease, the patient was referred to the local cardiothoracic center where he underwent an uncomplicated coronary artery bypass grafting.

## Discussion

Kawasaki disease is a form of systemic vasculitis affecting small- and medium-sized arteries typically occurring in childhood. Although usually self-limited, if it is left untreated it can have significant sequelae including coronary artery aneurysm in up to 20% of patients, highlighting the importance of early diagnosis [2]. The diagnostic criteria of KD in its acute phase are outlined in Table 1 [3].

**Table 1:** Criteria for diagnosis of KD. KD, Kawasaki disease

The diagnosis of KD requires the presence of fever lasting at least five days without any other explanation combined with at least four of the five following criteria	
1	Bilateral bulbar conjunctival injection
2	Oral mucous membrane changes including injected or fissured lips; injected pharynx or strawberry tongue
3	Peripheral extremity changes, including erythema of palms or soles, oedema of hands or feet (acute phase) and periungual desquamation (convalescent phase)
4	Polymorphous rash
5	Cervical lymphadenopathy (at least one lymph node >1.5 cm in diameter)
If ≥4 of the above criteria are present, diagnosis of KD can be made on day 4 of illness	

If KD is treated in the acute phase with aspirin at a dose of 80-100 mg/kg in four daily divided doses in addition to IV immunoglobulin, the incidence of coronary artery aneurysm can be reduced to 5% [4].

A retrospective study by Burns et al. noted that the mean age at presentation with late cardiovascular sequelae was 24.7 +/- 8.4 years, with symptoms including chest pain/myocardial infarction (60.8%), arrhythmia (10.8%), and sudden death (16.2%) [5]. The autopsy findings of deceased patients in this study showed coronary occlusion. It is prudent, therefore, to follow up patients with cardiovascular complications of KD. There are several modalities available that can be used to diagnose and monitor coronary aneurysms in vivo. Echocardiography is useful in the diagnosis of coronary abnormalities and has the added benefit of being able to assess myocardial function. In uncomplicated cases, this should be performed at diagnosis and then at intervals of one to two weeks and four to six weeks [5]. In patients who have known coronary aneurysms following acute KD, CT coronary angiography should be performed to monitor progression [6]. Recent studies have also shown that its results are comparable to catheter angiography.

In patients presenting with acute coronary syndrome, as in our case, invasive coronary angiography should be performed, and a diagnosis of KD can be made if findings of coronary calcifications and proximal aneurysms are identified. However, the exact dimensions of lesions can be difficult to assess on angiography as they may have significant thrombus burden or high calcium content. Therefore, intravascular ultrasound should ideally be employed to fully appreciate the actual dimension of these lesions before any intervention to ensure the correct size stent is employed for a given lesion [6-8].

A recent study has also looked at the potential for use of MRI in the diagnosis and surveillance of coronary lesions in KD [9]. This showed that MRI was able to identify wall motion abnormality as well as echocardiography and was superior to it in terms of identifying coronary aneurysms. However, MRI was inferior to angiography in terms of identifying stenoses and calcification. Although it may not give as much information as angiography in some ways, it has the advantage of being noninvasive and without any radiation exposure. MRI may prove to be a promising modality in the diagnosis and surveillance of patients with cardiac complications of KD.

Management of KD patients presenting with coronary syndromes differs from the patient without aneurysmal arteries. As the coronary arteries tend to be heavily calcified and rotational atherectomy should be performed before deployment of stents. Even then, experience with stents is limited in KD patients [7]. Bypass grafting may be considered in patients with inducible ischemia (as patients without this are best managed medically), and this should be undertaken for the same indications as in the general population. Bypass grafting has been shown to be superior compared to percutaneous coronary intervention, with patients undergoing PCI requiring repeat-revascularization more often as compared to patients undergoing coronary artery bypass grafting (CABG) [10-11].

## Conclusions

In younger patients presenting to hospital with acute coronary syndrome, KD should be considered as an underlying cause. This can be identified by angiography and by thorough questioning looking into history of unexplained pyrexia in childhood. The finding of aneurysms alone is not necessarily an indication for intervention with stents and medical treatment in the first instance should be pursued. If, however, there is evidence of inducible ischemia or patients present with acute coronary syndromes with the same indications for surgery or percutaneous intervention as may be found in the general population, then these treatment options should be pursued.
